# Deep learning-based predictive classification of functional subpopulations of hematopoietic stem cells and multipotent progenitors

**DOI:** 10.1186/s13287-024-03682-8

**Published:** 2024-03-13

**Authors:** Shen Wang, Jianzhong Han, Jingru Huang, Khayrul Islam, Yuheng Shi, Yuyuan Zhou, Dongwook Kim, Jane Zhou, Zhaorui Lian, Yaling Liu, Jian Huang

**Affiliations:** 1 Department of Mechanical Engineering and Mechanics, Lehigh University, Bethlehem, PA USA; 2 Coriell Institute for Medical Research, Camden, NJ USA; 3 Shanghai Key Laboratory of Medical Epigenetics, Laboratory of Cancer Epigenetics, Institutes of Biomedical Sciences, Medical College of Fudan University, Chinese Academy of Medical Sciences, Shanghai, People’s Republic of China; 4 Department of Bioengineering, Lehigh University, Bethlehem, PA USA; 5 Health and Human Biology, Brown University, Providence, RI USA; 6 Cooper Medical School of Rowan University, Camden, NJ USA; 7 Center for Metabolic Disease Research, Temple University Lewis Katz School of Medicine, Philadelphia, PA USA

**Keywords:** Hematopoietic stem cells, Multipotent progenitors, Deep learning, Evi1 protein, α-Catulin protein, GFP, Fluorescence-activated cell sorting

## Abstract

**Background:**

Hematopoietic stem cells (HSCs) and multipotent progenitors (MPPs) play a pivotal role in maintaining lifelong hematopoiesis. The distinction between stem cells and other progenitors, as well as the assessment of their functions, has long been a central focus in stem cell research. In recent years, deep learning has emerged as a powerful tool for cell image analysis and classification/prediction.

**Methods:**

In this study, we explored the feasibility of employing deep learning techniques to differentiate murine HSCs and MPPs based solely on their morphology, as observed through light microscopy (DIC) images.

**Results:**

After rigorous training and validation using extensive image datasets, we successfully developed a three-class classifier, referred to as the LSM model, capable of reliably distinguishing long-term HSCs, short-term HSCs, and MPPs. The LSM model extracts intrinsic morphological features unique to different cell types, irrespective of the methods used for cell identification and isolation, such as surface markers or intracellular GFP markers. Furthermore, employing the same deep learning framework, we created a two-class classifier that effectively discriminates between aged HSCs and young HSCs. This discovery is particularly significant as both cell types share identical surface markers yet serve distinct functions. This classifier holds the potential to offer a novel, rapid, and efficient means of assessing the functional states of HSCs, thus obviating the need for time-consuming transplantation experiments.

**Conclusion:**

Our study represents the pioneering use of deep learning to differentiate HSCs and MPPs under steady-state conditions. This novel and robust deep learning-based platform will provide a basis for the future development of a new generation stem cell identification and separation system. It may also provide new insight into the molecular mechanisms underlying stem cell self-renewal.

**Supplementary Information:**

The online version contains supplementary material available at 10.1186/s13287-024-03682-8.

## Background

Hematopoietic stem cells (HSCs) and multipotent progenitors (MPPs) are important for lifelong blood production and are uniquely defined by their capacity to self-renew while contributing to the pool of differentiating cells. HSC is a rare population in mouse bone marrow, with approximately 1 in 10^5^ cells being a transplantable HSC [1]. As HSCs differentiate, they give rise to a series of progenitor cells that undergo a gradual fate commitment to mature blood cells [2, 3]. Numerous studies have defined phenotypic and functional heterogeneity within the HSC/MPP pool and have revealed the coexistence of several HSC/MPP subpopulations with distinct proliferation, self-renewal, and differentiation potentials [4, 5]. Based on their self-renew capability, they can be divided into long-term (LT) and short-term (ST) HSCs, and multipotent progenitors (MPPs). In the adult mice, all HSCs/MPPs (HSPCs) are contained in the Lineage^−/low^Sca-1^+^c-Kit^+^ (LSK) fraction of the bone marrow (BM) cells [6]. Higher levels of HSC purity can be achieved by using signaling lymphocyte activation molecule (SLAM) family markers CD150 and CD48 [7]. It has been reported that one out of every ~ 2 LSK CD150^+^CD48^−^ cells possess the capability to give long-term repopulation in the recipients of BM transplants. Meanwhile, short-term HSCs (ST-HSCs) and MPPs can be isolated by sorting LSK/CD150^−^CD48^−^ and LSK/CD150^−^CD48^+^ cells, respectively [7]. As an alternative, HSCs can also be subdivided by CD34 and CD135 (FLT3) expression profiles. LSK/CD34^−^CD135^−^ cells are enriched with LT-HSCs, whereas LSK/CD34^+^CD135^−^ with ST-HSCs and LSK/CD34^+^CD135^+^ with MPPs [8]. So far, there is no evidence that those three subpopulations are morphologically distinguishable under light microscope.

Of note, several intracellular proteins, e.g., α-catulin and ecotropic viral integration site-1(Evi1), have been identified as functional markers in murine HSCs [9, 10]. Thus, GFP expression driven by α-catulin or *Evi1* gene promoters in mice has been utilized to identify HSCs and track their “stemness” in vivo or ex vivo [9–11].

Accumulating evidence has demonstrated that the HSC aging process is accompanied by functional decline. Specifically, HSCs from aged animals (aged HSCs) manifest an increase in immunophenotypic HSC number and a decrease in regenerative capacity compared to their counterparts from young animals (young HSCs). In addition, aged HSCs tend to differentiate more to the myeloid lineage over the lymphoid lineage, with decreased homing and increased polarity, epigenetic changes, and clonal expansion [12–14].

Deep learning (DL) has become the state of the art for many computer vision tasks in biomedical research [15, 16]. Supervised DL builds a mathematical model based on training samples with ground-truth labels. It extracts relevant biological microscopic characteristics from massive image data. The primary algorithm for DL image classification is based on the convolutional neural network (CNN). CNN is mainly composed of convolutional layers that perform a convolution with “learnable” filters. The parameters of these filters can be optimized during the learning process [16, 17]. Of note, previous studies have demonstrated that CNN can be used to predict stem cell fate [18–20].

**Fig. 1:**
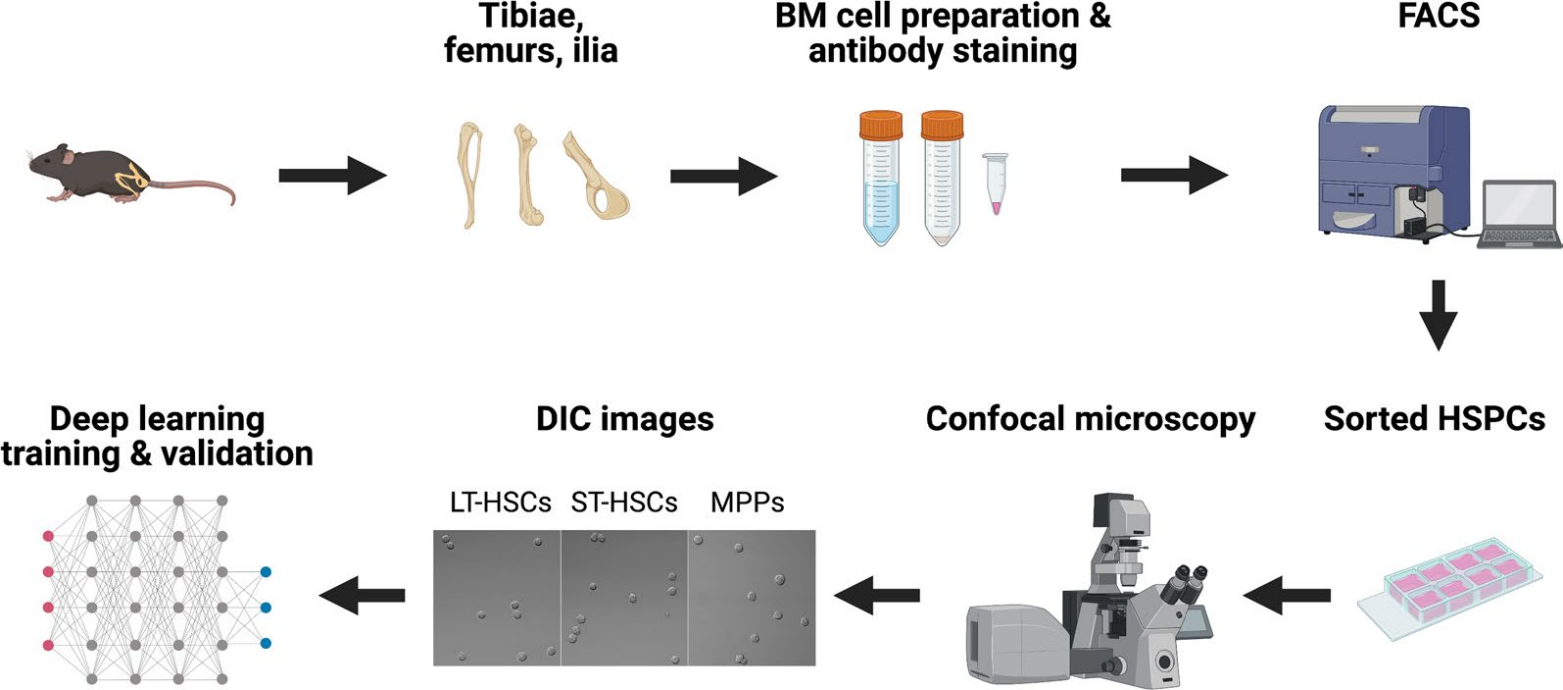
An overview of the flow of the experiment. The workflow depicts the steps from murine BM cell preparation, HSPCs isolation, image acquisition, to DL training and validation (Created with BioRender.com)

In our previous work, we have successfully developed a novel DL-based platform to detect rare circulating tumor cells with high accuracy [21]. In the present study, we investigated the potential of using DL to differentiate HSCs and MPPs based only on their morphology. First, we used a large dataset of Differential Interference Contrast (DIC) microscopy images of HSCs and MPPs to train the DL model, then assessed its efficacy with validation datasets (Fig. 1). After the DL model was established, we further tested it with HSCs and MPPs that were identified and isolated with different cell surface or intracellular makers. Our study demonstrated for the first time that deep learning can distinguish different subpopulations of hematopoietic precursors based on cell morphology. This novel and robust deep learning-based platform provided a proof-of-principle that an antibody-free, fluorescence/laser-free system is feasible to identify different cell populations purely based on cell morphology. It may also shed light on the molecular mechanisms underlying stem cell self-renewal.

## Methods

### Animals

C57BL/6(CD45.2), C57Bl/6-Boy/J(CD45.1) and α-catulin^GFP^ mice were purchased from the Jackson Laboratory. Evi1-IRES-GFP knock-in mice (Evi1^GFP^ mice) were kindly provided by Dr. Mineo Kurokawa at the University of Tokyo [10]. All mice were used at 8–12 weeks of age, except some Evi1^GFP^ mice were sacrificed at 24-month-old. Both male and female mice were used, and age matched. Each experimental group will include at least 4 mice. They were bred and maintained in the animal facility at Cooper University Health Care. All procedures and protocols were following NIH-mandated guidelines for animal welfare and were approved by the Institutional Animal Care and Use Committee (IACUC) of Cooper University Health Care.

### Antibodies

The following antibodies were used: mouse lineage cocktail-PE (BioLegend, cat# 78035), mouse lineage cocktail-APC (R&D Systems, cat# FLC001A), c-Kit-FITC (BioLegend, cat# 161603), c-Kit-APC (BioLegend, cat# 135108), c-Kit-PE/Cy7 (BioLegend, cat# 105814), c-Kit-BV421 (BioLegend, cat# 135124), Sca-1-BV605 (BioLegend, cat# 108133), Sca-1-APC (eBioscience, cat# 17-5981-82), Sca-1-BV421 (BioLegend, cat# 108127), Sca-1-PerCP-Cy5.5 (eBioscience, cat# 45-5981-82), CD150-BV421 (BioLegend, cat# 115925), CD150-PE (eBioscience, cat# 12-1501-82), CD150-PE-Cy7 (BioLegend, cat# 115913), CD48-PE/Cy7 (eBioscience, cat# 25-0481-80), CD48-BV711 (BioLegend, cat# 103439), CD48-APC-Cy7 (BioLegend, cat# 103432),CD34-FITC (eBioscience, cat# 11-0341-82), CD135-PE-Cy5 (BioLegend, cat# 135311), CD135-APC (BioLegend, cat# 135310), CD45.1-PerCP-Cy5.5 (BioLegend, cat# 110727), CD45.2-BV421 (BioLegend, cat# 109831), CD45.2-FITC(eBioscience, cat# 11-0454-82), Gr1-PE (BioLegend, cat# 108407), CD11b-APC (BioLegend cat# 101211), CD4-PE (BioLegend,cat# 116005), CD8a-PE (BioLegend, cat# 100707), B220-APC (BioLegend, cat# 103211).

### Flow cytometric analysis and cell sorting

Murine BM cells were flushed out from the long bones (tibias and femurs) and ilia with DPBS without calcium or magnesium (Corning). After lysis of red blood cells and rinse with DPBS, single-cell suspensions were stained with fluorochrome-conjugated antibodies at 4 °C for 15–30 min. Flow cytometric analysis and cell sorting were performed on a Sony SH800Z automated cell sorter or a BD FACSAria™ III cell sorter. Negative controls for gating were set by cells without antibody staining. All data were analyzed by using either the accompanying software with the Sony sorter or FlowJo software (v.10).

### DIC image acquisition and GFP fluorescence measurement

Fluorescence-activated cell sorting (FACS)-sorted cells were plated in coverglass-bottomed chambers (Cellvis) and maintained in DPBS/2% FBS throughout image acquisition. An Olympus FV3000 confocal microscope was used to take DIC and fluorescence images simultaneously at a resolution of 2048 × 2048. Fluorescence images of different cell groups were taken under exact the same recording conditions and GFP fluorescence intensities were measured by using Fiji software.

### Data processing

We built a MATLAB toolbox for image processing based on our previous work [21]. We used the toolbox to detect single cells in DIC images and remove the outliers (debris and cell clusters) by applying size thresholding and uniqueness checks. The toolbox then segmented the cells into cell-centered single-cell image crops of 64 × 64 pixels and labeled them by cell types. We applied data augmentation to the training examples using arbitrary image transformation including random rotation, horizontal flipping, and brightness adjustment on the original single-cell crops. We practiced oversampling on the minor classes in each run during the training experiment to balance different training samples. The oversampling algorithm randomly sampled training images from the minority until the number of the examples reached the same number in the majority class. Therefore, in our experiment, the training dataset for each run contained equivalent numbers of data samples for all three classes.

### Deep learning framework and training

Our deep learning models utilized the ResNet-50 architecture [22] as the pretrained layers that were fine-tuned by the training datasets. ResNet-50 is a convolutional neural network (CNN) architecture that is commonly used for image classification tasks. It consists of 5 convolutional blocks with varying numbers of convolutional layers in each block. The convolutional layers in ResNet-50 extract features from input images at different levels of abstraction, with the deeper blocks learning more complex features. ResNet-50 also includes shortcut connections through skip connections that add the input to the output of the convolutional layers. This facilitates better feature learning by maintaining a strong gradient flow during training. Following the convolutional blocks, our models had two fully-connected layers with Rectified Linear Unit (ReLu) activation functions and a dropout layer with a dropout rate of 0.3 to prevent overfitting during training. The models used a SoftMax activation function with a cross-entropy loss for generating predicted results. The ADAM optimizer with a weight decay of 0.05 was applied for training experiments, with a learning rate of 5 × 10^–4^ for the fully-connected layers and a retraining of the convolutional layers at 1% of the learning rate. We trained the model with a batch size of 512 for 20 epochs on a Tesla P100 GPU on the Google Colab platform with Pytorch 1.10.0. The final training outcome was reported with a training and validation split of 8:2.

### Long-term competitive reconstitution assays

The experiments were performed as previously described [23]. Briefly, adult congenic recipient mice (CD45.1) were lethally irradiated (1000 rad, split dose 3 h apart). Purified donor cells were then injected along with 3 × 10^5^ wild type “competitor” cells (CD45.1) into the retro-orbital plexus of individual recipient mice. To induce anesthesia for the retro-orbital injection, mice were exposed to Isoflurane to minimize discomfort and stress, administered through inhalation for a duration of 30–40 s. For the euthanasia of mice, it was performed using CO_2_ inhalation followed by cervical dislocation, in strict adherence to the American Veterinary Medical Association (AVMA) guidelines. Hematopoietic reconstitution was monitored over time in the peripheral blood using conjugated antibodies to CD45.2 (104, FITC), B220 (6B2), Mac-1 (M1/70), CD3 (KT31.1), and Gr-1 (8C5). All recipient mice (*n* = 3 each group) were sacrificed after 4 months and their BM cells were collected and stained with the following: Lineage cocktail-PE, CD45.1-PerCP-Cy5.5, CD150-PE-Cy7, c-Kit-APC, CD48-APC-Cy7, and CD45.2-BV421, Sca-1-BV605.

### Statistical analysis

Data are presented as means ± SEM unless otherwise stated. The statistical significance was determined by the one-way ANOVA (for experiments with multiple groups) or the unpaired two-tailed Student’s *t* test (for two groups comparison). *p < 0.05; **p < 0.01; ***p < 0.001.

## Results

### Preparation of HSPC subpopulations and image datasets for DL training

**Fig. 2:**
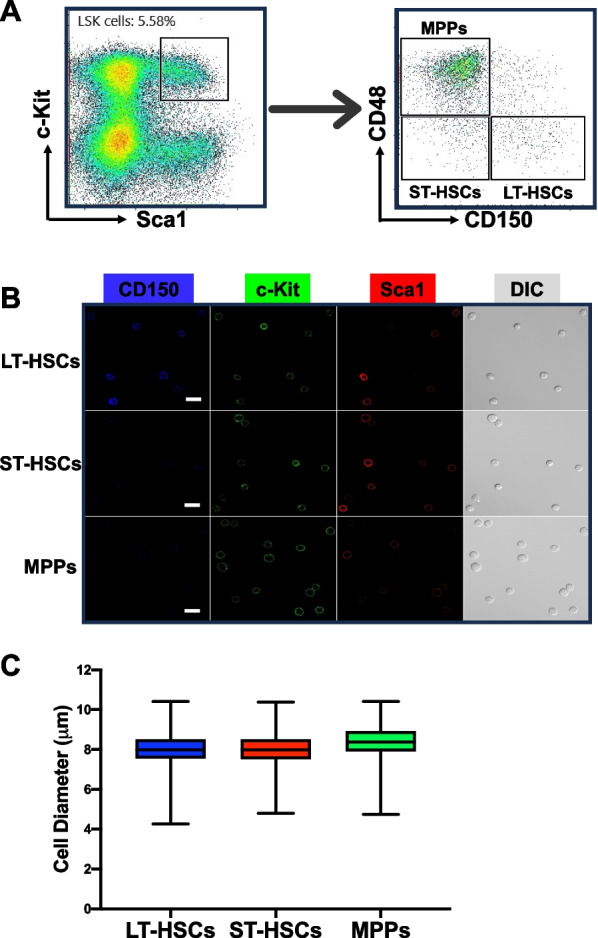
LT-HSCs, ST-HSCs, and MPPs do not exhibit significant difference in size. **A** Representative FACS density dot plots show the gating strategy employed to identify and isolate LT-HSCs, ST-HSCs, and MPPs from murine BM. **B** DIC and fluorescence images were taken immediately after FACS. Typical images are shown. Scale bar (in white) = 10 μm. **C** The box plot depicting the cell diameter dispersion of LT-HSCs, ST-HSCs, and MPPs. The boxes represent the middle 50% (interquartile range, IQR) of cells and the solid lines inside the boxes are the medians. The upper and lower whiskers indicate the cells that are outside the middle 50% range, and they are calculated as ± 1.5 × IQR. Outliers are not shown. *n* = 5

To explore whether we could use DL to distinguish different subsets of HSPCs based on their morphology, we first isolated HSCs and MPPs from murine BM by FACS. We used a well-established combination of surface markers consisting of LSK (lineage^−^Sca1^+^c-Kit^+^) and SLAM (CD150 and CD48) markers and sorted out three subpopulations: LT-HSCs (LSK/CD150^+^CD48^−^), ST-HSCs (LSK/CD150^−^CD48^−^) and MPPs (LSK/CD150^−^CD48^+^) (Fig. 2A). We then seeded those cells in culture chambers with coverglass bottoms and acquired DIC and confocal fluorescence images (Fig. 2B). Over 96% of the recorded cells in the images exhibit anticipated fluorescence features (Fig. 2B), indicating that the sorting process was accurate and reliable. In DIC images, most cells (~ 95%) have a spherical shape (Fig. 2B) while the rest are irregular or polymorphic. The cell membranes of these cells appear to be rough, but no specific morphological features unique to any cell population can be identified through visual inspection. LT-HSCs, ST-HSCs and MPPs are small cells, majority of which have a diameter less than 10 μm. Figure 2C shows the dispersion of the cell sizes of these cells. Large cell outliers make up approximately 0.10% of the MPPs and 0.03% of the two HSC groups. Our measurement shows that the average diameters (mean ± SEM) of LT-HSCs, ST-HSCs, and MPPs are 8.05 ± 0.02, 8.05 ± 0.03, and 8.44 ± 0.02 μm, respectively, and they are not significantly different.

### Development of a novel DL model to distinguish LT-HSCs, ST-HSCs, and MPPs

To build a DL model to distinguish murine HSCs and MPPs, we first utilized a customized MATLAB toolbox to automatically locate individual cells in acquired DIC images and segmented them into cell-centered single-cell image crops of 64 × 64 pixels. The image crops were labeled by cell types as ground truth. From five independent experiments, we compiled an image dataset for DL model training and validation, comprising 4050 LT-HSCs, 7868 ST-HSCs, and 9676 MPPs. We applied data augmentation to enhance data diversity and avoid overfitting, practiced oversampling to balance the significance of the minority subsets, and employed transfer learning to obviate the need for bigger datasets (detailed information described in “Materials and Methods”).

**Fig. 3:**
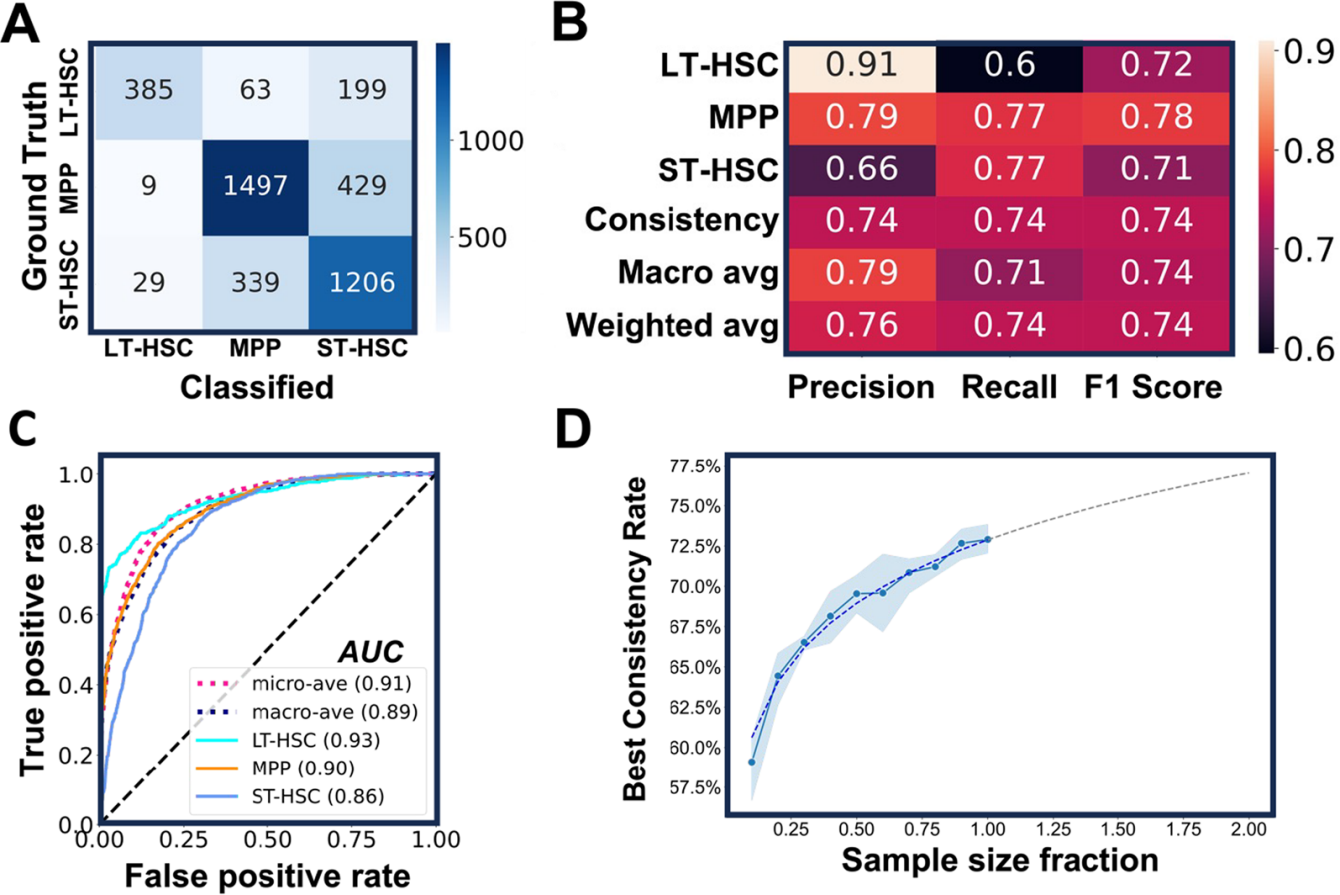
The LSM model's performance in predicting distinct hematopoietic precursor subpopulations. **A** The confusion matrix of the LSM model. After the training and validation process, new cells from three HSPC subsets were classified by the model and the results are summarized. *n* = 5. **B** The performance metrics of the model. **C** The ROC-AUC curve of the model. All AUC values are above 0.85, indicative of a good performance of the model. Diagonal dashed line represents a random classifier with no discrimination. **D** The performance of the LSM model is correlated with the training sample size. With increased sample sizes (from 10 to 100% of total training data), the overall consistency rate of the model improved accordingly. For each sample size, five iterations of training were executed with 20 epochs. The mean of the overall consistency rate (blue dots) and the corresponding 95% confidence interval (blue shade) are shown. The dashed line is a fitted curve with extrapolation

We designed the new DL model as a three-class classifier that would assign three probability scores to every cell tested. The scores are between 0 and 1 with the sum of three scores equal to 1. The predicted cell type is determined by the highest probability score (prediction score) that ranges from 0.34 to 1. After several rounds of training and validation, the DL model was challenged with the cells it had never seen before. The results are summarized in a confusion matrix (Fig. 3A). Out of 647 FACS-sorted LT-HSCs, 60% were classified as LT-HSCs, 30% as ST-HSCs and 10% as MPPs. Therefore, the rate of consistency between the DL classification and the immunophenotypic sorting is 60% for LT-HSC group. Similarly, the consistency rates for ST-HSC and MPP were 77% (1206/1574) and 77% (1497/1935), respectively. Based on these results, various measures were generated to gauge the performance of the method (Fig. 3B), including precision (positive predictive value), recall (sensitivity), macro average (arithmetic mean), and weighted average (average adjusted by sample sizes). Our DL model achieved an overall F1 score of 0.74 (Fig. 3B) and high area under the curve of the receiver operating characteristic (ROC-AUC) scores (Fig. 3C), which suggests that the model was performing well in the classification of LT-HSCs, ST-HSCs, and MPPs. This model will henceforth be referred to as the LSM model (L stands for LT-HSCs, S for ST-HSCs, and M for MPPs).

Generally, more data input results in better DL model prediction. We therefore investigated how the size of a dataset would influence the performance of the LSM model. To this end, we first randomly sampled 80% of the previous training dataset (17,438 cells in total) to serve as the full-scale training sample (13,950 cells) and used the remainder (3488 cells) for validation. We divided the training sample randomly into 10 fractions (1395 cells each fraction). While keeping all other essential parameters constant, we trained the model with incremental sample fractions until the entire training sample was used. We performed 5 iterations for each training sample size, and at the end of each training, validation dataset was classified, and the overall consistency rate was used to gauge the performance of the LSM model (Fig. 3D). As anticipated, the size of training samples is positively correlated with the performance of the LSM model, however, the correlation is not linear (Fig. 3D). When 80% of the training samples were used, the overall consistency rate (71%) was nearly as good as what could be achieved with the entire training samples (73%). An extrapolation based on the real data points predicts that the overall consistency rate can approach 77% if the training sample size is doubled (Fig. 3D). These data indicate that the LSM model can be further improved. However, an experiment as we just described may be needed to decide whether the benefits of model improvement worth the time, effort, and cost required to expand the dataset.

### The LSM model differentiates cells based on their morphological features

**Fig. 4:**
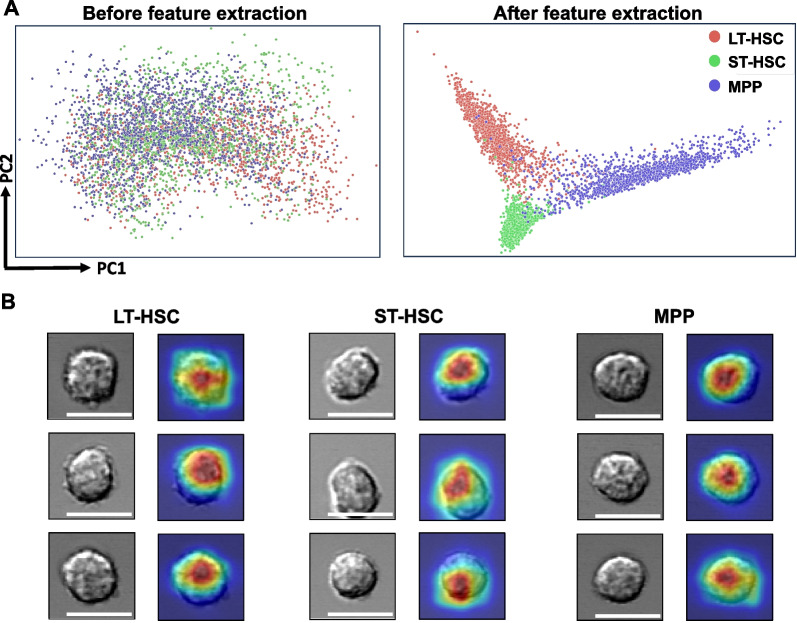
Interpretation of the LSM model. **A** Cell imaging data were processed by principal component analysis and the PCA score plots show the striking difference that feature extraction by the LSM model can make. **B** Visual explanation of the LSM model. Cells from the three groups were randomly selected and their attention heatmaps in the LSM model were generated by Score-CAM. Red regions received the highest attention by the LSM model, while blue regions were largely ignored. *n* = 5. Scale bar = 10 μm$.$

After multiple rounds of training and validation, the LSM model obtained the capability to differentiate HSCs and MPPs. To elucidate what the LSM model had learned from this process, we first performed a principal component analysis (PCA) on the cell images. PCA reduced the high-dimensional information from the original imaging data into two-dimensional principal components (PC1 and PC2). On the PCA plot, the distribution of HSCs and MPPs is dispersed and mixed (Fig. 4A, left), indicating that these cell types were not distinguishable from each other at this moment. In comparison, after being processed by the LSM model, imaging data were analyzed in the same way. Strikingly, cell type specific clusters were formed with limited overlap (Fig. 4A, right). These results proved that the LSM model can extract cell-specific morphological features from different cell types. Next, we constructed a class activation map (Score-CAM) from the convolutional layers of the LSM model (Fig. 4B). Score-CAMs are commonly used to explain how a DL model learns to classify an input image into a particular class [24]. On a heat map, the regions receiving strong attention from the DL model are colored in red, while blue color means the areas are ignored. When the LSM model was given the single-cell image inputs, its strongest attention was attracted to the areas that were almost exclusively within the cell boundaries (Fig. 4B). Taken together, our data indicate that cell morphology captured in the light microscopy images contains crucial information for accurately classifying cells by the LSM model.

### The cellular features extracted by the LSM model are intrinsic to HSCs

**Fig. 5:**
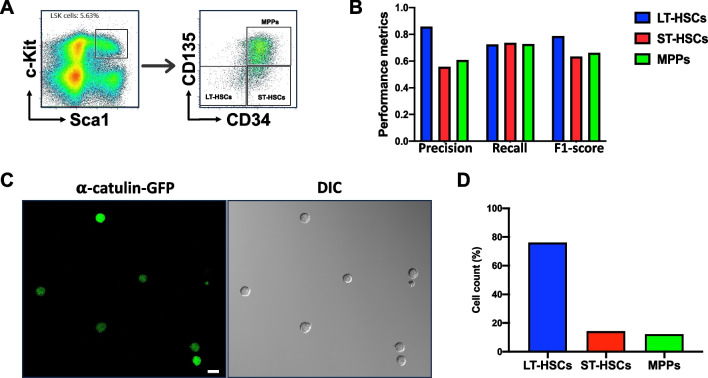
The LSM model distinguishes HSPCs sorted with LSK/CD34/CD135 surface markers and α-catulin-GFP. **A** Representative FACS density dot plots show the gating strategy employed to sort murine LT-HSCs, ST-HSCs, and MPPs using LSK/CD34/CD135 surface markers. **B** The performance metrics of the LSM model in the classification of HSPCs obtained from **A**. A total of 4142 LT-HSCs, 873 ST-HSCs, and 1780 MPPs were obtained from 4 C57BL/6 mice and tested. **C** DIC and fluorescence images of LSK/α-catulin-GFP^+^ cells were taken immediately after FACS. Representative images are shown. Scale bar = 10 μm. **D** Total 1227 LSK/α-catulin-GFP^+^ cells were obtained from 4 α-catulin^GFP^ mice and analyzed by the LSM model. 74% of them were classified as LT-HSC

In previous experiments, HSPCs were identified and isolated based on the binding of antibodies to corresponding surface antigens. It is possible that certain antibody-antigen interactions may result in cell-specific morphological manifestation, which could make the efficacy of the LSM model antibody/antigen dependent. To exclude this possibility, we first tested the LSM model with the HSPCs that were sorted out based on LSK/CD34/CD135, another set of surface markers widely used to identify and isolate murine HSPCs [8]. The immunophenotypes of LT-HSCs, ST-HSCs, and MPPs were shown in Fig. 5A. Although the F1 scores for the classification of ST-HSC and MPP are slightly lower than those seen previously, the performance of the LSM model on LT-HSC classification remains consistent (Fig. 5B). It’s noteworthy that under current condition, the immunophenotypic LT-HSCs are CD34/CD135 double negative. These data suggest that the crucial cell-specific information for accurately classifying cells by the LSM model is not likely to derive from the antibody/antigen interactions.

We further addressed the issue by utilizing the α-catulin protein, an intracellular marker of HSCs that has been found to be expressed almost exclusively in murine HSCs [9]. In the α-catulin^GFP^ mice, α-catulin-GFP^+^c-Kit^+^ cells in the BM are mainly LT-HSCs with a small portion of ST-HSCs [9]. We therefore first sorted out LSK/α-catulin-GFP^+^ cells (Fig. 5C), and then used the LSM model to classify the cells. A total of 1227 LSK/α-catulin-GFP^+^ cells were classified. In line with expectation, 74% of them were classified as LT-HSC, 14.0% as ST-HSC, and 12% as MPP (Fig. 5D). Together, our data indicate that neither antibody/antigen interaction nor GFP overexpression has a significant impact on the classification of cells using the LSM model, particularly in the context of LT-HSC classification. What the LSM model learned is intrinsic to the tested cells, i.e., LT-HSCs, ST-HSCs and MPPs.

### The LSM model can prospectively identify murine functional HSCs

**Fig. 6:**
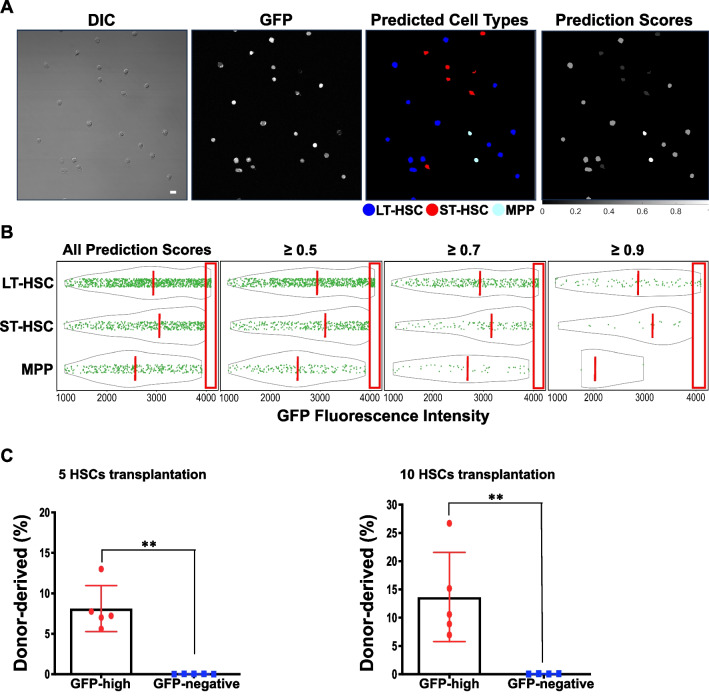
Long-term competitive reconstitution of HSCs based on prospective classification by the LSM model. **A** LSK/Evi1-GFP^+^ cells were FACS-sorted from Evi1^GFP^ transgenic mice and classified by the LSM model. A representative field of view is shown. Scale bar = 10 μm. **B** GFP fluorescence analysis of the classified cell types. Solid green dots are individual GFP^+^ cells and the gray outlines depict the distribution of their GFP fluorescence intensity. The orange dashed lines indicate the medians of each cell groups. Increasing the prediction score threshold from 0.34 (all scores) to 0.9 didn’t significantly change fluorescence intensity features among predicted cell types, and the cells with the highest GFP expression (inside red boxes) were always classified as LT-HSC. *n* = 5. **C** Competitive reconstitution of irradiated mice with GFP-high cells. Top 3% high GFP expressing cells were FACS-sorted from the LSK/Evi1-GFP^+^ pool and transplanted into lethally irradiated recipients. Each recipient was transplanted with 5 or 10 GFP-high cells and 3 × 10^5^ host-derived BM cells. GFP-negative LSK cells were used as control. BM was harvested after 4 months for chimerism analysis. *n* = 3 each group. **p < 0.01 (compared to GFP-negative group. Unpaired *t* test)

It has been demonstrated that the LSM model is capable of differentiating isolated HSPC subpopulations. We then asked how it would perform prospectively in a mixture of HSPCs without the use of SLAM or CD34/CD135 surface markers. To answer this interesting question, we challenged the LSM model with LSK/GFP^+^ BM cells from the Evi1^GFP^ transgenic mice, another animal model in HSC studies [10]. Evi1 is a transcription factor of the SET/PR domain protein family and plays a critical role in maintaining HSC stemness [10]. Unlike the α-catulin^GFP^ transgenic mice, GFP expression in the Evi1^GFP^ mice is controlled by Evi1 gene promoter, and it’s found in over 90% immunophenotypic LT-HSCs, ~ 80% ST-HSCs, and ~ 30% MPPs [10]. Therefore, LSK/Evi1-GFP^+^ cells are a mixture of HSPCs. Out of the 1726 LSK/Evi1-GFP^+^ cells classified by the LSM model, 55% were predicted as LT-HSC, 27% as ST-HSC, and 18% as MPP (Fig. 6A). A fluorescence analysis revealed that GFP expression in all three predicted cell types varied greatly, however, strong GFP expression was more frequently seen in the predicted HSCs (Fig. 6B, left). In line with this finding, the average GFP fluorescence intensity of all predicted HSCs was higher than that of the predicted MPPs (Fig. 6B, left), which is consistent with a previous report [10]. This trend was not affected when the prediction score threshold (manifesting the confidence of the LSM model) was increased to 0.5, 0.7, or 0.9 (Fig. 6B, right). Although GFP expression patterns were similar in predicted HSCs, average GFP intensity was slightly higher in the predicted ST-HSC population (Fig. 6B). Among the cells that were tested, a minority (~ 6%) exhibited the highest level of GFP (GFP-high). Importantly, all those cells were predicted by the LSM model as LT-HSC.

It has recently been shown that in early embryonic development, high Evi1 expressing cells are predominantly localized to the intra-embryonic arteries and preferentially give rise to HSCs [25]. Based on this report and the prediction of the LSM model, we proposed that high Evi1 expressing precursors in adult murine BM are true functional HSCs. To test this hypothesis, we conducted a competitive transplantation experiment using FACS-sorted top 3% GFP-high cells from the LSK/Evi1-GFP^+^ pool, with GFP negative LSK cells as the control. We transplanted 5 or 10 GFP-high or GFP-negative cells (CD45.2) along with 3 × 10^5^ wildtype (CD45.1) “competitor” cells into lethally irradiated recipient mice (CD45.1). After 4 months, we harvested BM from the transplanted mice and measured chimerism (percentage of donor-derived cells). As shown in Table 1, the numbers of chimeric-positive mice—defined by convention as > 1% donor-derived (CD45.2) cells in either BM or peripheral blood—were significantly higher in GFP-high group (5/5 mice for 5 cells and 5/5 for 10 cells). In contrast, no long-term reconstitution was found in GFP-negative group (0/5 and 0/4 for 5 cells and 10 cells). The degrees of chimerism for GFP-high 5-cell group (mean = 8.116%) and 10-cell group (mean = 13.67%) were substantially higher than those for the GFP-negative 5-cell (mean = 0.035%) and 10-cell group (mean = 0.064%) (Fig. 6C). These results suggested that the LSM model has the potential to prospectively identify functional murine HSCs.

**Table 1:** Competitive reconstitution of irradiated mice with GFP-high cells

Donor cell population	Numbers of donor cells injected per recipient mice	Numbers of recipient mice	Recipient mice with multilineage reconstitution
BM LSK/Evi1-GFP high	5 cells	5	100% (5/5)
	10 cells	5	100% (5/5)
BM LSK/Evi1-GFP negative	5 cells	5	0% (0/5)
	10 cells	4	0% (0/4)

### Deep learning cannot differentiate MPP subpopulations (MPP2-4) from their DIC images

Accumulating evidence indicates that MPPs can be further divided into at least three subpopulations (MPP2-4), which exhibit different lineage bias and functions [26]. To test whether DL can be used to differentiate MPP subpopulations, we tried to build a new 3-class classifier exclusively for MPP classification. We adopted the same strategy for model training and validation when feeding the DL platform with DIC image dataset of different MPPs (Additional file 1: Fig. S1). After several trials, the consistency rate of classification was much lower than the LSM model. Particularly, after we introduced more convolutional layers in ResNet (see “Methods” for details), the performance of the model didn’t improve, suggesting a bottleneck had been reached. By comparison, the LSM model worked very well in the classification of all three MPP subpopulations (Additional file 2: Table S1). These results suggest that deep learning, as a powerful cell classification tool, has its limitations and its success depends on target cells. On the other hand, these data proved again that the LSM model is a reliable classifier for general MPP identification.

### Deep learning can differentiate immunophenotypically identical aged and young HSCs

**Fig. 7:**
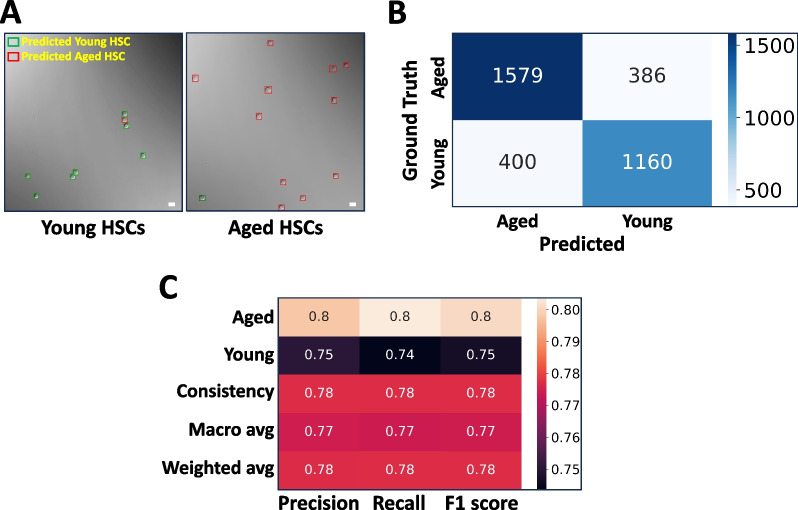
The YA model differentiates young and aged LT-HSCs. **A** The YA model was trained to differentiate LT-HSCs from young and aged mice using the image data of immunophenotypic LT-HSCs (LSK/SLAM/GFP^+^). Typical model predictions in DIC images are shown. Scale bar = 10 μm. **B** After the YA model was trained, it was tested with new image data sets. The results are summarized in the confusion matrix. *n* = 5 each group. **C** The performance metrics of the YA model

It is well known that HSCs from aged mice (aged HSCs) are functionally defective compared with their counterparts in young mice (young HSCs), though they have the same immunophenotypes (LSK/CD150^+^CD48^−^) on flow cytometry. We were wondering whether the functional difference had any manifestation in their morphology. To investigate this issue, we designed a new model based on the previous DL platform. In brief, we FACS-sorted out LT-HSCs (LSK/CD150^+^CD48^−^) from the BM of young (8–10 weeks old) and aged (24 months old) mice, and then compiled DIC image datasets. After training, validation, and optimization, the new DL model was able to separate the two populations accurately and is herein named as the YA model (Fig. 7). First and foremost, the YA model viewed most young HSCs (74%) as one type of cell and the majority of aged HSCs (80%) as another, though both cells were LSK/CD150^+^CD48^−^. Interestingly, a small percentage of young HSCs (26%) were classified as aged HSC, and vice versa in aged HSCs (20% were classified as young HSC) (Fig. 7A, B). The overall F1 score of the YA model is 0.78 (Fig. 7C), which is higher than the LSM model.

## Discussion

Hematopoietic stem and progenitor cells (HSPCs) are a critical component of bone marrow (BM) transplants, which are a mainstay of life-saving therapy for patients with leukemia and congenital blood disorders. Currently, FACS is the primary method for identifying and separating HSPCs. While powerful, it has several drawbacks: it requires antibody staining and laser light sources to produce scattered and fluorescent signals, which can negatively affect cell viability and stem cell activity [27]. In contrast, a deep learning-based platform has the potential to be developed into a label-free and laser-free method for HSPC studies with further technical improvements. In this study, we provided evidence to support the concept that long-term HSCs (LT-HSCs), short-term HSCs (ST-HSCs), and multipotent progenitors (MPPs) can be classified in a steady state using the deep learning method. Interestingly, the intrinsic cell-specific information needed for effective identification can be obtained from light microscopy images alone. Additionally, our deep learning model can differentiate between functionally distinctive young HSCs and aged HSCs, which have the same immunophenotypes [12–14]. Without performing a tedious long-term limiting dilution transplantation assay, it is currently impossible to distinguish these cells. The success of our young vs aged (YA) model supports the idea that deep learning can be developed into a unique tool for assessing the functional states and activities of HSCs. In conjunction with or without flow cytometry, deep learning is expected to have more applications in the study of HSPCs, especially when it is integrated with various imaging and cell separation hardware systems.

The heterogeneity of immunophenotypically sorted HSCs and MPPs is well documented. For instance, long-term competitive reconstitution assay confirms less than 50% of LT-HSCs identified by LSK/SLAM markers [7]. Likewise, the frequency of true LT-HSCs in LSK/α-catulin-GFP^+^ is only 33% [9]. Therefore, while evaluating the performance of our deep learning models, we did not consider FACS-sorted cell populations as an absolute gold standard and refrained from using the terms "accuracy" or "accuracy rate". Instead, we opted for the term "consistency rate" to indicate that we were comparing two different methods designed for the same purpose.

Although flow cytometry-based HSC separation is a well-established technique, our deep learning model can provide value in various contexts. Firstly, as it is well documented, the phenotype of stem cells can change developmentally [28] or when regeneration is stimulated by agents such as 5-fluorouracil [29], making it challenging to identify HSCs based on surface markers alone. Additionally, surface markers may also change during HSC culture and expansion [30]. In such circumstances, our morphology-based identification method may become important for accurately identifying HSCs.

An intriguing question that remains unanswered is which morphological features are crucial for our deep learning models to make accurate classifications. This is a complex but fundamental issue for deep learning as an analyzing method. Deep learning is a powerful tool that can extract various cell features, such as morphology, granularity, biomass, and more [17, 31]. For instance, deep learning can be trained to detect and measure cell size and shape in microscopic images, which can help identify abnormalities or changes in cell morphology [32, 33]. In this study, we carefully registered the sizes and shapes of the target cells (Fig. 2C) to evaluate their impact on our model's classifications. As there was no significant difference in size and shape between LT-HSCs, ST-HSCs, and MPPs, we postulate that these parameters did not play a crucial role in the cell classifications made by our models. It has been reported that in addition to steady state morphology, dynamic cellular behaviors in artificial experimental settings can serve as multidimensional datasets for deep learning to learn and extract [34], which may reflect the cellular difference in intracellular protein concentration and localization. However, it is not clear what specific features were extracted by deep learning in those experimental settings. It’s noteworthy that our effort to differentiate MPP subtypes by deep learning failed, which not only reflects the limitations of deep learning but also implies that the learning and extraction process could be highly cell specific.

Lastly, it's worth noting that our LSM model's performance has been tested in hematopoietic precursors harvested from at least 33 mice, and the results have shown high consistency. However, we still need to separate different HSC populations based on our LSM model and conduct a stringent functional transplantation to validate the efficacy of stem cell identification and classification through this technology.

## Conclusions

While our work is still in its early phases, it has not only broadened the application of deep learning but also provides a promising avenue for uncovering previously unknown features of HSCs. This approach has significant potential to advance our understanding of stem cell biology.

### Supplementary Information

**Additional file 1**. **Fig. S1**. Flow Cytometry Scheme for Distinguishing Various Murine MPP Subpopulations. Representative FACS density dot plots show the gating strategy employed to identify and isolate MPP2, MPP3, and MPP4 from murine BM.

**Additional file 2**. **Table S1**: Classification of MPP subpopulations using the LSM model.

## Data Availability

All data needed to evaluate the conclusions in the paper are present in the paper and/or the Supplementary Materials. We are in the process to deposit and share our raw image data into the Medical Imaging and Data Resource Center (MIDRC), which is supported by the National Institute of Biomedical Imaging and Bioengineering (NIBIB).

## Abbreviations

HSCs: Hematopoietic stem cells
HSPC: Hematopoietic stem and progenitor cells
LT-HSCs: Long-term hematopoietic stem cells
ST-HSCs: Short-term hematopoietic stem cells
MPPs: Multipotent progenitors
BM: Bone marrow
DIC: Differential interference contrast
SLAM: Signaling lymphocyte activation molecule
LSK: Lineage^−^/lowSca-1^+^ c-Kit^+^
Evi1: Ecotropic viral integration site-1
DL: Deep learning
CNN: Convolutional neural network
FACS: Fluorescence-activated cell sorting
ReLu: Rectified linear unit
ROC-AUC: Area under the curve of the receiver operating characteristic
PCA: Principal component analysis
