# Supplementary figures and images for: Deep learning-based predictive classification of functional subpopulations of hematopoietic stem cells and multipotent progenitors
Source: Stem Cell Res Ther. 2024 Mar 13;15:74. doi: 10.1186/s13287-024-03682-8 (PMC10935795; doi:10.1186/s13287-024-03682-8)

**Fig. S1. FACS of different murine MPP subpopulations.**

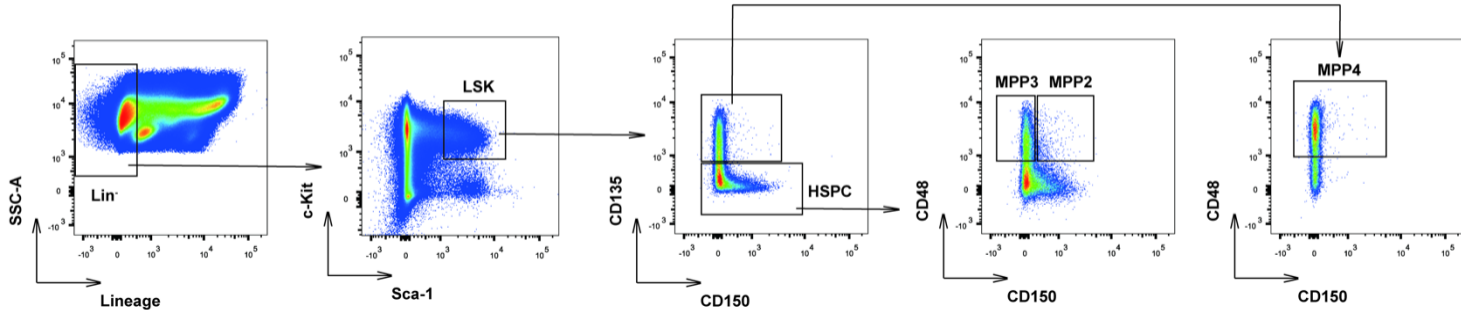

Supplement: Supplementary file 1 — Additional file 1. Fig. S1. Flow Cytometry Scheme for Distinguishing Various Murine MPP Subpopulations. Representative FACS density dot plots show the gating strategy employed to identify and isolate MPP2, MPP3, and MPP4 from murine BM. [file 13287_2024_3682_MOESM1_ESM.pdf]
