# Supplementary material for: Deep learning-based predictive classification of functional subpopulations of hematopoietic stem cells and multipotent progenitors
Source: Stem Cell Res Ther. 2024 Mar 13;15:74. doi: 10.1186/s13287-024-03682-8 (PMC10935795; doi:10.1186/s13287-024-03682-8)
Supplement: Supplementary file 2 — Additional file 2. Table S1: Classification of MPP subpopulations using the LSM model. [file 13287_2024_3682_MOESM2_ESM.pdf]

Table S1: Classification of MPP subpopulations using the LSM model.

		Consistency Rate of Classification Under Different Prediction Score Threshold		
MPP Subpopulations	Numbers of Cells Tested	≥ 0.34	≥ 0.50	≥ 0.70
MPP2	477	90% (428/477)	98% (392/399)	100% (277/277)
MPP3	1490	90% (1346/1490)	97% (1229/1262)	99% (813/815)
MPP4	1301	90% (1170/1301)	97% (1059/1091)	99% (726/727)
